# PROSPECTIVE ASSOCIATION OF INTRINSIC CAPACITY WITH FALLS AND MULTIPLE FALLS IN OLDER ADULTS (NHATS)

**DOI:** 10.1093/geroni/igad104.3539

**Published:** 2023-12-21

**Authors:** Kewei Shi, Lingxiao He, Xiaodong Chen, Ya Fang

**Affiliations:** Xiamen University, Xiamen, Fujian, China (People’s Republic); Xiamen University, Xiamen, Fujian, China (People’s Republic); Xiamen University, Xiamen, Fujian, China (People’s Republic); Xiamen University, Xiamen, Fujian, China (People’s Republic)

## Abstract

**Background:**

Intrinsic capacity (IC) is a recently proposed novel metric of evaluating the overall health condition of older people. The IC has been associated with multiple adverse health outcomes while its associations with falls and multiple falls in community-dwelling older adults are not well studied.

**Methods:**

2113 community-dwelling older adults (aged 68-97 years) were selected from two waves of the National Health and Aging Trends Study (NHATS). Data of five IC domains (cognitive, sensory, locomotion, vitality, psychological), falls and multiple falls were collected via questionnaires at baseline and a 4-year follow-up. The IC was evaluated as an IC score calculated by averaging the Z-score in each IC domain. Logistic regression was used to analyze the associations between falls and IC scores with controls for age, gender, marital status, comorbidity index and social activity index.

**Results:**

A higher baseline IC score was associated with a lower risk of falls (OR= 0.459, 95%CI [0.309,0.681]) and multiple falls (OR= 0.278, 95%CI [0.154, 0.502]). Participants with smaller IC score change demonstrated greater risk of fall (OR= 0.512, 95%CI [0.336, 0.777]) and multiple falls (OR= 0.238, 95%CI [0.128, 0.435]). In terms of interactive effects, there were also no significant interactions between baseline IC score and IC score change in their associations with fall outcomes.

**Conclusions:**

The IC is longitudinally associated with falls and multiple falls in older adults. Our findings emphasize the potential role of IC as a valuable marker for predicting fall risk in the older population.

